# **A novel** ***Aureobasidium pullulans*** **isolate for the biological control of** ***Penicillium commune*** **in potato decay and its optimization for enhanced antifungal activity using the Taguchi method**

**DOI:** 10.1007/s10123-026-00863-8

**Published:** 2026-07-17

**Authors:** Noushin Shafiee, Keivan Beheshti-Maal, Hamid Reza Akkafi, Hashem Nayeri

**Affiliations:** 1 Department of Microbiology, Faculty of Biological Sciences, Fal. C, Islamic Azad University, Falavarjan, Isfahan, Iran; 2 Department of Plant Science, Faculty of Biological Sciences, Fal. C, Islamic Azad University, Falavarjan, Isfahan, Iran; 3 Department of Biochemistry, Faculty of Biological Sciences, Fal. C, Islamic Azad University, Falavarjan, Isfahan, Iran

**Keywords:** *Aureobasidium pullulans*, Biocontrol activity, Extracellular antifungal metabolites, *Penicillium commune*, Taguchi

## Abstract

*Penicillium commune* is a significant postharvest fungal pathogen responsible for blue mold rot in stored potatoes, resulting in substantial economic losses worldwide. In this study, we isolated and characterized a novel epiphytic strain of *Aureobasidium pullulans* SBAN-IAUF-1 from potato foliage and evaluated its antagonistic activity against *Penicillium commune* SBAN-IAUF-12. The new isolate exhibited strong inhibitory effects, reducing pathogen growth by 60% in vitro and 69.41% in vivo, outperforming values reported for most previously studied *A. pullulans* strains. Extracellular antifungal metabolites produced by *Aureobasidium pullulans* SBAN-IAUF-1 also showed substantial antifungal activity, indicating that extracellular antifungal metabolites play a key role in antagonism. To further enhance extracellular antifungal metabolites activity, we applied a Taguchi L9 orthogonal design, which identified optimal culture conditions and significantly increased the extracellular antifungal metabolites activity. The optimal conditions suggested by L9 analysis for maximizing extracellular antifungal metabolites production were 20 °C, 7 days of incubation, pH 5, and 1% (wv^− 1^) YMB concentration. This study provides the first report of a potato-derived *A. pullulans* isolate optimized through Taguchi methodology for controlling *Penicillium commune*. The strong biocontrol activity of *Aureobasidium pullulans* SBAN-IAUF-1 highlights its potential as an environmentally safe and effective candidate for postharvest management of blue mold disease in potatoes.

## Introduction

Potatoes rank fourth among globally imported crops, following rice, wheat, and corn (Ibrahim et al. 2014). Currently, the worldwide area under potato cultivation is approximately 164,000 hectares (Dadrasi et al. 2022). This crop holds substantial economic importance and is expected to make a significant contribution to global nutrition in the years to come. However, its yield is markedly reduced by various diseases. Potato tubers are susceptible to numerous diseases, and their identification, prevention, and management are essential for enhancing large-scale potato production (Fahim Abbas et al. 2013; Shah et al. 2023). Fungi are among the primary agents of postharvest potato diseases, responsible for considerable economic losses worldwide. Pathogens such as *Rhizopus stolonifer*, *Aspergillus flavus*, *Fusarium oxysporum*, *Fusarium ambucaine*, *Fusarium solani*, *Fusarium graminearum*, *Phytophthora infestans*, *Alternaria solani*, *Alternaria grandis*, *Alternaria alternata*, as well as various *Penicillium* and *Verticillium* species, have been frequently isolated from decayed potato tubers (Ibrahim et al. 2014; Shi et al. 2023).

The genus *Penicillium*, belonging to the division *Ascomycota*, is a pathogenic group of fungi responsible for blue mold disease in numerous food crops, including cereals, legumes, corn, fruits, and vegetables. This genus comprises nearly 300 species. For instance, *Penicillium solitum* produces enzymes such as polygalacturonase, which contribute to cell wall degradation and pathogenicity in plants (Shah et al. 2023). In potatoes, blue mold initially enters the tubers through wounds and subsequently infects the internal tissues (Kim et al. 2008). Although chemical fungicides are widely used for disease control due to their low cost and effectiveness, their continuous application has resulted in the emergence of resistant fungal strains, carcinogenic risks, fetal toxicity, and environmental contamination. Consequently, there is an urgent need for alternative strategies to manage postharvest potato diseases (Zhu et al. 2019; Zhang et al. 2019).

Microbial biocontrol is regarded as an effective alternative to chemical fungicides. Moreover, many antagonistic microorganisms isolated from fruit surfaces exhibit strong environmental adaptability and may serve as superior biocontrol agents compared to other microbial antagonists (Di Francesco et al. 2021). Yeasts, in particular, offer several advantages as biocontrol agents, including rapid growth in fermenters using inexpensive substrates, high tolerance to environmental stresses such as temperature, humidity, and oxygen fluctuations, and the ability to colonize fruit surfaces for extended periods (Ferraz et al. 2016; Sanzani et al. 2021).

The use of biocontrol yeasts against phytopathogenic molds was first proposed by Jacobs and Van Vuuren (1991), and since then, numerous studies have reported their effectiveness in managing plant pathogens. Walker et al. (1995) demonstrated that strains of *Pichia anomala*, *Pichia membranifaciens*, and *Saccharomyces cerevisiae* can inhibit the growth of various fungal phytopathogens. Weiler and Schmitt (2003) reported significant suppression of *Fusarium oxysporum* by *Zygosaccharomyces bailii*. Additionally, Santos and Marquina (2004) showed that *Botrytis cinerea*, the causal agent of gray mold disease in grapes, can be effectively controlled by *P. membranifaciens* (Santos et al. 2004; Rosa et al. 2010).

The ability of yeasts to colonize the surfaces of fruits and vegetables arises from their capacity to utilize host nutrients without causing pathogenic effects. Yeast colonization on these surfaces creates both nutritional and spatial competition with plant pathogens and can stimulate the production of defense-related enzymes in the host (Zhu et al. 2019). Moreover, unlike bacteria and filamentous fungi, yeasts do not produce allergenic spores, mycotoxins, or antibiotics (Edward-Rajanayagam et al. 2023).

In general, the biocontrol mechanisms of yeasts include competition for nutrients and space, secretion of non-volatile extracellular metabolites such as enzymes, toxins, and siderophores, production of volatile organic metabolites, and the induction of resistance responses in plants (Freimoser et al. 2019).

The Taguchi method is a statistical approach used for designing and analyzing experiments to identify optimal conditions while requiring a minimal number of trials. Instead of modifying one variable at a time, this method enables the simultaneous evaluation of multiple factors. One of its major advantages is that, beyond determining optimal conditions, it quantifies the relative influence of each factor, such as temperature, pH, or nutrient sources, and identifies settings that are more stable and less sensitive to fluctuations. Ultimately, the Taguchi method reduces the number of necessary experiments, thereby lowering both cost and time, while efficiently identifying the key variables and defining the most effective conditions to achieve the desired outcome (Hisam et al. 2024).

The present study aimed to isolate and screen biocontrol yeasts, investigate the biocontrol mechanisms of the most effective yeast against native fungal pathogens isolated from potatoes, and optimize its antifungal activity against pathogenic molds using the Taguchi statistical approach. Although *Aureobasidium pullulans* has been reported as a biocontrol agent in numerous studies, isolates from potato agroecosystems remain poorly understood, and the optimization of their antifungal metabolites has been rarely examined. In this study, we introduced *A. pullulans* SBAN-IAUF-1 as a new potato-derived isolate with strong inhibitory activity against *Penicillium commune*. Using a Taguchi L9 design, we improved the production and effectiveness of its extracellular antifungal metabolites. These results show the potential of *A. pullulans* SBAN-IAUF-1 as a promising and environmentally friendly option for postharvest control of potato diseases.

## Materials and methods

### Chemicals and culture media

The culture media used in this study included PDA medium (potato, 200 g L⁻¹; dextrose, 20 g L⁻¹; agar, 20 g L⁻¹), YEPD-MB medium (yeast extract, 10 g L⁻¹; peptone, 20 g L⁻¹; dextrose, 20 g L⁻¹; agar, 20 g L⁻¹; methylene blue, 0.03 g L⁻¹), YEPD broth (yeast extract, 10 g L⁻¹; peptone, 20 g L⁻¹; dextrose, 20 g L⁻¹), YEPD agar (yeast extract, 10 g L⁻¹; peptone, 20 g L⁻¹; dextrose, 20 g L⁻¹; agar, 20 g L⁻¹), and YMB medium (yeast extract, 3 g L⁻¹; malt extract, 3 g L⁻¹; peptone, 5 g L⁻¹; dextrose, 10 g L⁻¹).

### Isolation and macroscopic and microscopic identification of the potato fungal pathogen

Rotten potato tubers were collected from local storage facilities and farm in Isfahan, Iran, and were washed with distilled water and dried using alcohol-soaked cotton. Subsequently, 5 × 5 mm sections were excised from the diseased tissues with a sterile scalpel and cultured on PDA medium (Ibrahim et al. 2014). The plates were incubated at 25 °C for 72 h. Mold colonies growing on PDA were repeatedly subcultured until distinct and purified colonies were obtained (Shah et al. 2023). The purified mold isolates were then examined for their macroscopic and microscopic characteristics (Agu and Chidozie 2021).

### Koch’s principles for confirming the pathogenicity of the isolated fungus

Healthy potato tubers were surface-sterilized with 0.2% (vv^-1^) sodium hypochlorite for 15 min, rinsed with distilled water, and dried under sterile conditions. A small hole was made on one side of each tuber using a sterile toothpick, and 20 µL of a conidial suspension (1 × 10⁶ conidia mL⁻¹) of the fungus was injected into the wound. The inoculated tubers were placed in plastic bags, and 5 mL of sterile distilled water was added to the bottom of each bag to maintain humidity. The bags were then kept at 20 °C for three weeks. After symptoms appeared, the fungus was re-isolated and purified again to confirm its pathogenicity according to Koch’s postulates (Bhunjun et al. 2021). This experiment was performed in three replicates.

### Molecular identification of the isolated fungus

Genomic DNA from the mold was extracted using the phenol–chloroform method using a previously described method (Erami et al. 2023). DNA amplification was carried out using the universal primers ITS1 and ITS4. The PCR reaction (20 µl total volume) contained 10 µl of PCR master mix, 6 µl of sterile distilled water, 1 µl of forward primer, 1 µl of reverse primer, and 2 µl of template DNA. The PCR program included an initial denaturation at 95 °C for 5 min, followed by 35 cycles of 94 °C for 30 s, 56 °C for 45 s, and 72 °C for 45 s, with a final extension at 72 °C for 5 min. The size of the PCR product was confirmed by agarose gel electrophoresis, and the purified products were sent to Pishgam Biotechnology Company for sequencing. The obtained sequences were aligned using NCBI GenBank to identify the fungal genus and species and were subsequently deposited in GenBank. A phylogenetic tree was constructed using the Neighbor-Joining (NJ) method implemented in MEGA software. Bootstrap analysis was used to evaluate the reliability of the inferred phylogenetic relationships (Aboutalebian et al. 2019).

### Isolation and screening of biocontrol yeasts

Sterile wet cotton swabs were used to sample the surfaces of potato leaves from a potato farm in Isfahan, Iran, and immediately placed into 100 mL Erlenmeyer flasks containing 50 mL of YEPD broth supplemented with 100 mg L⁻¹ ampicillin and 50 mg L⁻¹ chloramphenicol. The flasks were incubated at 28–30 °C for 24 h with shaking at 150 rpm. Aliquots were then plated on YEPD agar containing the same antibiotics. All plates were incubated at 28–30 °C for 48 h, and yeast colonies were repeatedly subcultured until pure cultures were obtained (Perez et al. 2016).

The killer activity of the yeast isolates was tested against the isolated mold. A conidial suspension of the mold, grown for 5 days, was prepared at a concentration of 1 × 10⁶ conidia mL⁻¹. Then, 100 µL of the suspension was spread onto YEPD-MB plates buffered at pH 4.3–4.7. Different yeast isolates were spotted onto the plates previously spread with the mold suspension using sterile toothpicks. The plates were incubated at 25 °C for 3–5 days, and the killer activity of each yeast was evaluated by the presence of an inhibition zone around the yeast spot (Moura et al. 2021).

### Molecular identification of the biocontrol yeast

The colony-PCR method was used to identify the biocontrol yeast. One single yeast colony was suspended in 50 µl of sterile distilled water, heated at 95 °C for 10 min, and then immediately cooled on ice. The suspension was centrifuged at 8000 g for 5 min, and 1 µl of the supernatant was used as the PCR template. DNA amplification was carried out using the universal primers F1R (5′-CATTAAATCAGTTATCGTTTATTTG-3′) and R1Y (5′- TGATACCCCCGACCGTCCC-3′) (Zolfaghari et al. 2024). The PCR conditions consisted of an initial denaturation at 94 °C for 5 min, followed by 35 cycles of 94 °C for 30 s, 48 °C for 40 s, and 72 °C for 50 s, with a final extension at 72 °C for 5 min. The PCR product was checked by agarose gel electrophoresis, and the amplified fragment was sent to Pishgam Biotechnology Company for sequencing. The obtained sequences were aligned using the NCBI GenBank database to determine the genus and species of the yeast, and then deposited in GenBank. A phylogenetic tree was constructed using the Neighbor-Joining (NJ) method implemented in MEGA software. Bootstrap analysis was used to evaluate the reliability of the inferred phylogenetic relationships (Walch et al. 2016).

### In vitro biocontrol activity assay

To prepare the treatment plates, a conidial suspension of the pathogenic mold (1 × 10⁶ conidia mL⁻¹) was first prepared. Then, 5 µL of this suspension was placed at the center of a PDA plate. The yeast isolate was inoculated at two points located 3 cm from the center. The control plates contained only PDA inoculated with 5 µL of the pathogenic mold suspension. All plates were incubated at 25 °C for 15 days (Perez et al. 2016). The experiment was performed in triplicate. The average colony diameter of the mold was measured in both control and treatment plates. The biocontrol activity of the yeast isolate was expressed as the Control Index (CI%), calculated using the formula described by Sanzani et al. (2021):

$$CI(\%)=(\frac{A-B}{A})\times100$$

where:

CI = control index.

A = mold colony area in the control.

B = mold colony area in the treatment.

### In vivo biocontrol activity assay

Healthy potato tubers were surface-sterilized with 0.2% (vv^-1^) sodium hypochlorite for 10 min and rinsed with sterile distilled water. Three holes were made in a row on each tuber using a sterile toothpick. Each hole was inoculated with 20 µL of the yeast suspension (1 × 10⁸ cells mL⁻¹), followed by 20 µL of the pathogen conidial suspension (1 × 10⁶ conidia mL⁻¹). Positive controls received only the pathogen, while negative controls were inoculated with either the yeast suspension or sterile distilled water. The positive control, negative control, and treatment groups were placed in separate plastic packages, with a small amount of sterile distilled water added to the bottom to maintain humidity. Each group consisted of three tubers. All packages were incubated at 20 °C for two weeks. After incubation, the average rot diameter around the inoculation holes was measured for both control and treatment groups, and the pathogen growth inhibition by the yeast isolates was calculated using the previously described formula (Mari et al. 2012). This experiment was performed in three replicates.

### Quantification of the antifungal activity of extracellular metabolites produced by yeast

This assay was conducted to verify the production of extracellular antifungal metabolites by the yeast. A loopful of a 48-h yeast culture was inoculated into 100 mL of YMB medium in a 250-mL Erlenmeyer flask and incubated at 25–30 °C with shaking at 150 rpm for 72 h. The culture was then centrifuged at 10,000 × g for 15 min at 4 °C, and the supernatant was filtered through a 0.45-µm membrane filter. Next, 100 µL of the sterile filtrate was added to 7-mm wells in YEPD-MB plates previously inoculated with 10⁶ conidia mL⁻¹ of the target mold. The plates were incubated at 20 °C for 72 h, after which the inhibition zone diameters were recorded. Antifungal activity was expressed in arbitrary units (AU), following the definition of Bajaj et al. (2013). One AU was defined as the amount of extracellular antifungal metabolites required to produce a 1 mm inhibition zone in the well diffusion assay. Therefore, AU values were determined directly from the measured diameter of the inhibition zone and expressed as arbitrary units. This experiment was performed in three replicates and three plates were used per treatment.

### Optimization of antifungal activity of extracellular metabolites produced by yeast using Taguchi design

Based on previous studies, four factors) temperature, incubation time, pH, and YMB concentration) were selected for optimization, each tested at three levels (Table 1) (Vero et al. 2009; Agirman and Erten 2020). Accordingly, nine experimental runs were generated using the Taguchi L9 design in Qualitek 4 software (Table 2). After performing the experiments under shaking conditions (150 rpm), the antifungal activity of the yeast-derived extracellular metabolites (expressed in AU) was quantified using the well-plate assay. The obtained data were then entered into Qualitek 4 software for statistical analysis. This experiment was performed in three replicates. After the experiments were conducted, the software analyzed and suggested the optimal conditions. The Taguchi design was analyzed using Qualitek-4 software based on the average response values obtained from replicated experiments. Optimization was conducted using the “Using Average of Results” option rather than the signal-to-noise (S/N) ratio approach. The quality characteristic was defined as “Bigger is Better”, and the optimal factor levels were selected according to the highest mean antifungal activity (AU) predicted by the software. The relative influence of the experimental factors was evaluated based on their percentage contribution and analysis of variance (ANOVA) results. Finally, a confirmation experiment was conducted under these conditions.

**Table 1:** Effective factors and levels in the extracellular antifungal activity using the Taguchi statistical design

Factors	Level 1	Level 2	Level 3
Temperature (°C)	20	25	30
Incubation time (d)	3	5	7
pH	4	5	6
YMB concentration (wv^− 1^) (%)	1	1.5	2

**Table 2:** L9 designed experiments to investigate the extracellular antifungal activity using Qualitek 4 software

Test Number	Temperature (˚C)	Incubation time (d)	pH	YMB concentration (wv^− 1^) (%)
1	20	3	4	1
2	20	5	5	1.5
3	20	7	6	2
4	25	3	5	2
5	25	5	6	1
6	25	7	4	1.5
7	30	3	6	1.5
8	30	5	4	2
9	30	7	5	1

### Statistical analyses

The Qualitek 4 uses its own ANOVA analysis. Differences between control and treatment groups were evaluated using Duncan’s multiple range test at a significance level of *P* < 0.05. All statistical analyses were performed using SPSS version 20.

## Results

### Isolation of the fungus and confirmation of its pathogenicity on potato

**Fig. 1:**
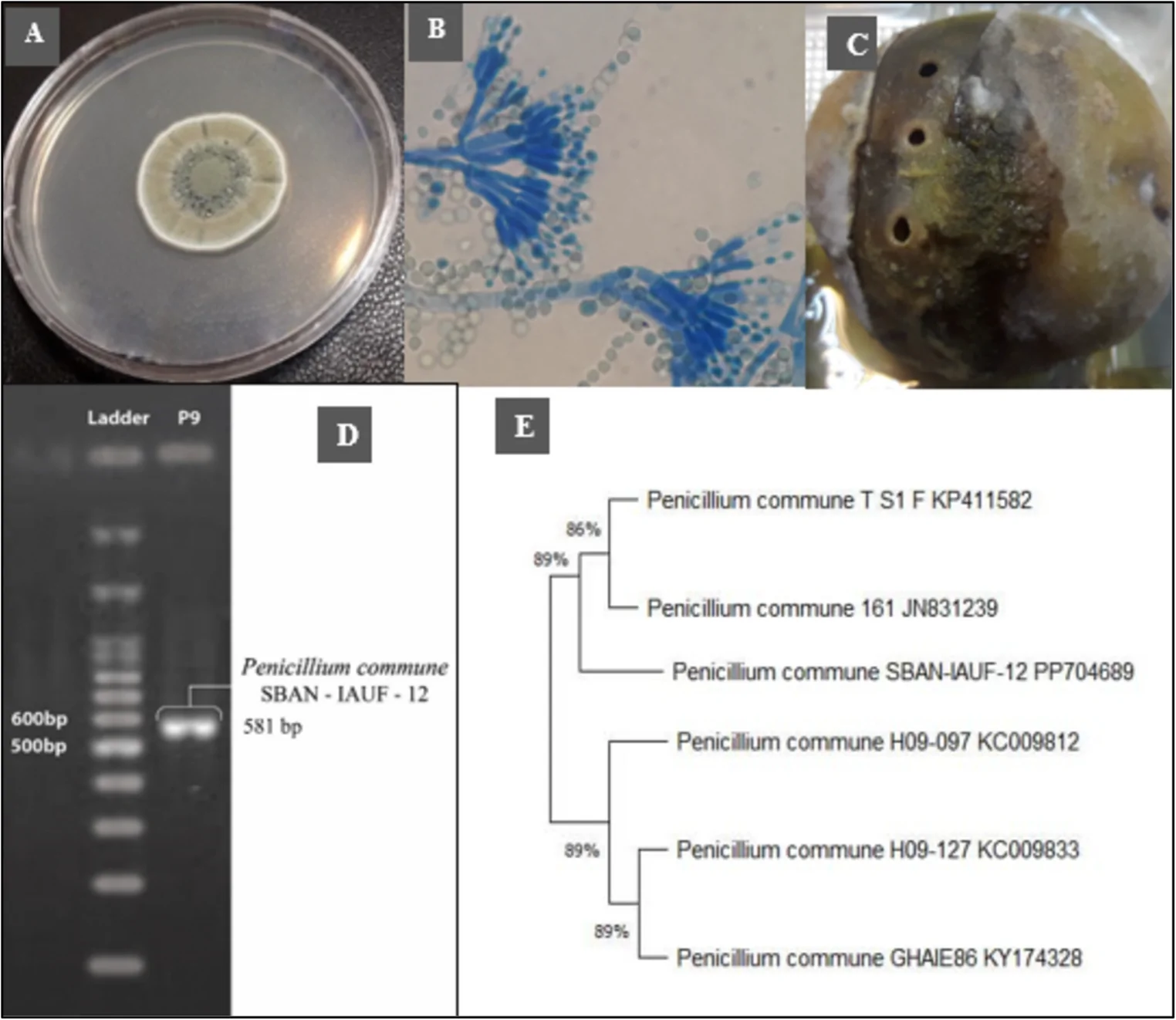
(**A**) Macroscopic appearance of the P9 isolate. (**B**) Microscopic morphology of the P9 isolate. (**C**) Pathogenicity assay on potato based on Koch’s postulates using the P9 isolate. (**D**) A 581 bp amplicon observed on agarose gel electrophoresis following amplification of the ITS region of the P9 isolate. (**E**) Phylogenetic tree of the ITS region of *P. commune* (accession number PP704689.1)

In this study, several mold strains were isolated from postharvest rotted potato tubers collected in Isfahan. Some isolates showed green pigmentation; one of these, designated P9, was selected for further analysis. Macroscopic and microscopic observations indicated that P9 belonged to the *Penicillium* genus. Its colonies were dark green and powdery with a clear white margin (Fig. 1A). Microscopically, the isolate produced broom-like conidiophores characteristic of *Penicillium* spp. (Fig. 1B).

Pathogenicity tests were performed following Koch’s postulates. Three weeks after inoculating potato tubers with the conidial suspension, the pathogenicity of P9 was confirmed. In *Penicillium*-induced potato rot, the tissue at the inoculation site first darkened, after which a white mycelial layer developed and gradually turned green and eventually dark (Fig. 1C). No specific odor was detected during this process. Re-isolation of the fungus on PDA and incubation for 72 h at 25 °C produced colonies identical to the original isolate, both macroscopically and microscopically, confirming that the recovered mold was the same *Penicillium* sp.

### Molecular identification of the potato fungal pathogen

Molecular methods were used to determine the species-level identity of the fungal isolate recovered from the potato. Electrophoresis of the PCR product, run alongside a 100 bp marker, showed that the amplicon from the ITS region was 581 bp in size (Fig. 1D). BLAST analysis in GenBank indicated that the sequence shared 98.96% similarity and 99% query coverage with *Penicillium commune* T_S1_F (KP411582.1). Phylogenetic analysis confirmed that strain SBAN-IAUF-12 clustered with *P. commune* reference strains, supporting its identification as *P. commune* (Fig. 1E). The isolate was subsequently deposited in GenBank as *P. commune* SBAN-IAUF-12 under accession number PP704689.1.

### Screening, macroscopic, and microscopic examination of biocontrol yeasts

**Fig. 2:**
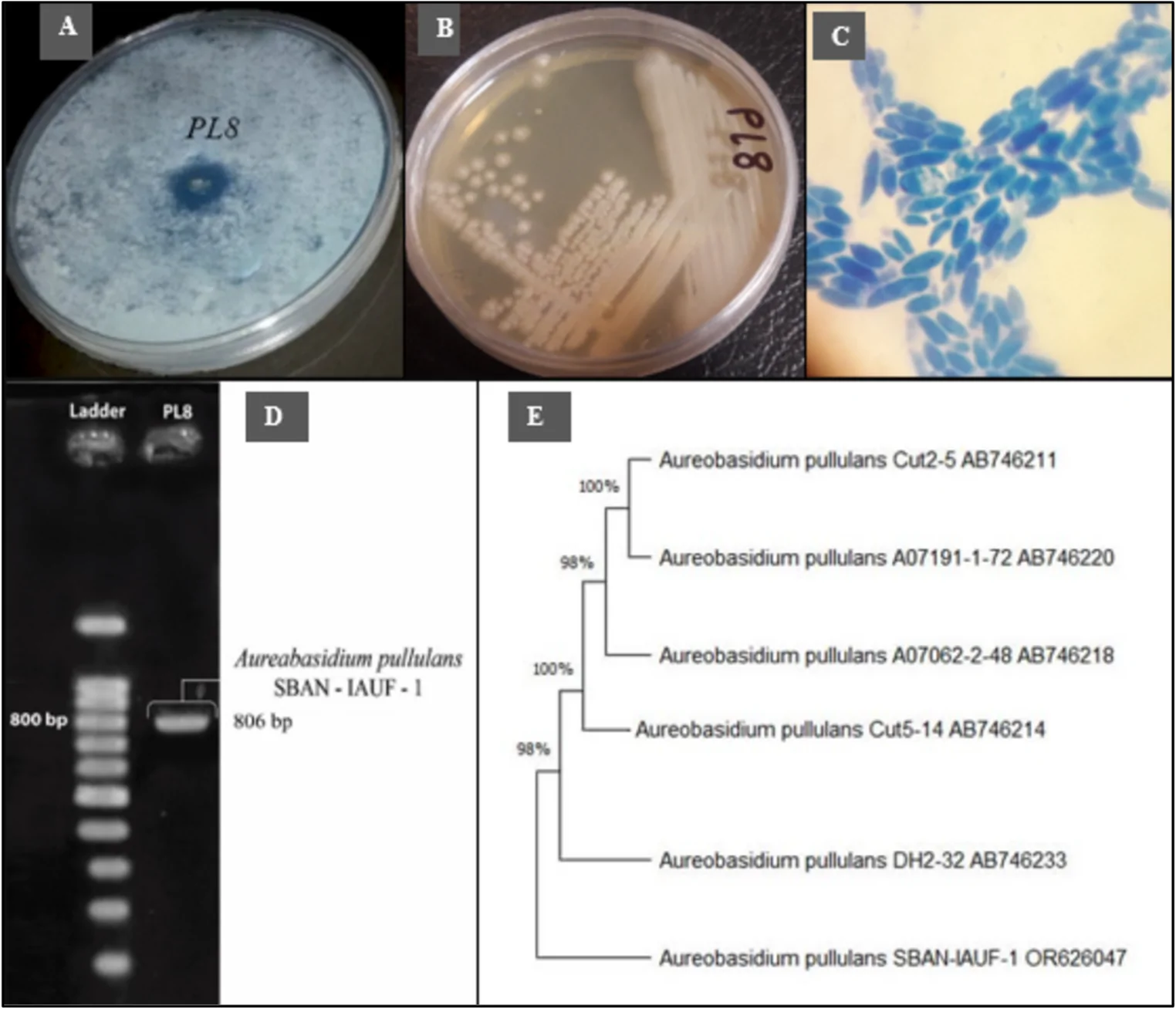
**A**: Killer activity assay of the PL8 isolate, showing a clear inhibition zone around the yeast spot against fungal cells. **B**: Macroscopic morphology of the PL8 isolate. C: Microscopic morphology of the PL8 isolate. **D**: An 800 bp amplicon observed in agarose gel electrophoresis following amplification of the *18 S-rDNA* region in the PL8 isolate. **E**: Phylogenetic tree of the *18 S-rDNA* sequence corresponding to *A. pullulans* SBAN-IAUF-1

Biocontrol screening based on inhibition zone measurements showed that the PL8 isolate, isolated from potato leaves, displayed the strongest antifungal activity among all tested yeast isolates (Fig. 2A). In macroscopic evaluations, PL8 produced large, soft colonies with a distinct central elevation (Fig. 2B). Microscopic examination at 100× magnification using Nikon Eclipse E100 microscope demonstrated that the cells of this isolate were elongated and spindle-shaped (Fig. 2C).

### Molecular identification of the biocontrol yeast

Agarose gel electrophoresis of the PCR product obtained from the PL8 isolate showed an 800 bp amplicon of the *18 S-rDNA* region generated using the universal primers F1R and R1Y (Fig. 2D). BLAST sequence analysis in GenBank indicated that the isolate shared 98.87% similarity and 99% query coverage with *Aureobasidium pullulans* DH2-32 (AB746233.1). Phylogenetic analysis confirmed that strain SBAN-IAUF-1 clustered with *A. pullulans* reference strains, supporting its identification as *A. pullulans* (Fig. 2E). The isolate was deposited in the NCBI GenBank under the name *Aureobasidium pullulans* SBAN-IAUF-1 (accession number OR626047).

### In vitro and in vivo biocontrol activity assay

**Fig. 3:**
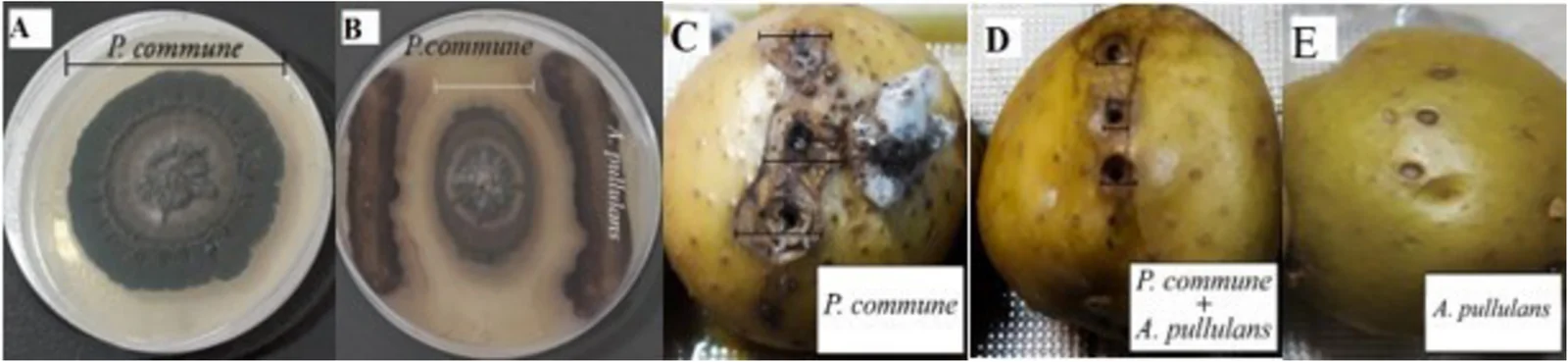
(**A**) Control plate: PDA medium inoculated only with *P. commune* SBAN-IAUF-12. (**B**) Treatment plate: PDA medium co-inoculated with *P. commune* SBAN-IAUF-12 and *A. pullulans* SBAN-IAUF-1. (**C**) Positive control: potatoes inoculated with *P. commune* SBAN-IAUF-12. (**D**) Treatment group: potatoes co-inoculated with *P. commune* SBAN-IAUF-12 and *A. pullulans* SBAN-IAUF-1. (**E**) Negative control: potatoes inoculated with *A. pullulans* SBAN-IAUF-1

The in vitro biocontrol activity of *A. pullulans* SBAN-IAUF-1 against *P. commune* SBAN-IAUF-12 was evaluated by measuring the diameter of fungal growth. The control and treatment plates are shown in Fig. 3A and B, respectively. The results demonstrated that *A. pullulans* SBAN-IAUF-1 inhibited the growth of the pathogenic mold by 60%.

The in vivo biocontrol activity of *A. pullulans* SBAN-IAUF-1 was also assessed. After 14 days, the mean rot areas in the positive control (Fig. 3C), treatment (Fig. 3D), and negative control (Fig. 3E) groups were 0.857, 0.26, and 0 cm^2^, respectively, showing statistically significant differences among groups. The detailed statistical analysis is presented in Table 3. Overall, the in vivo assays indicated that *A. pullulans* reduced potato rot caused by *P. commune* by 69.41%.

**Table 3:** Average rot area and ANOVA results for the potato groups: positive control (inoculated with *P. commune* SBAN-IAUF-12), treatment group (co-inoculated with *P. commune* SBAN-IAUF-12 and *A. pullulans* SBAN-IAUF-1), and negative control (inoculated with *A. pullulans* SBAN-IAUF-1)

Different groups of potatoes	Descriptive statistics results	ANOVA test results		
	Mean ± S.D.	F		Sig.
Control +	0.85 ± 0.21 a	9.834		0.005
Treatment	0.26 ± 0.06 b			
Control -	0.00 ± 0.00 b			

Similar letters indicate that the average numbers are the same.

Significance level: (α = 0.05)

### Quantification of extracellular antifungal activity

**Fig. 4:**
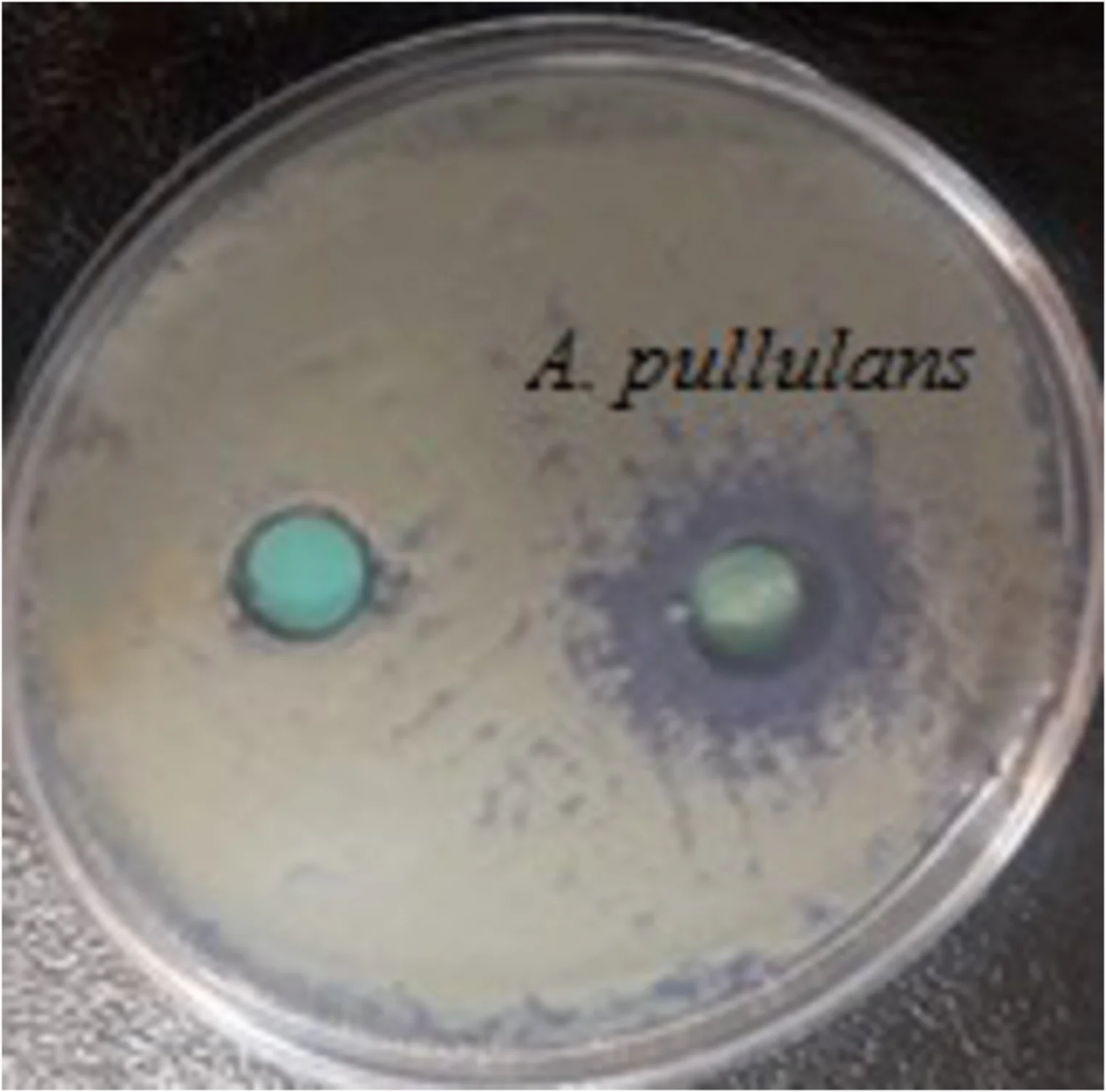
Clear inhibition zones formed around wells containing extracellular metabolites produced by *A. pullulans* SBAN-IAUF-1.

A clear inhibition zone around the well containing the filtered supernatant obtained from the *A. pullulans* SBAN-IAUF-1 culture confirmed that this yeast produces extracellular metabolites with antifungal activity against the pathogenic fungus (Fig. 4). The antifungal activity of these extracellular metabolites was calculated as 15 AU.

### Optimization of yeast biocontrol activity using the Taguchi experimental design

**Fig. 5:**
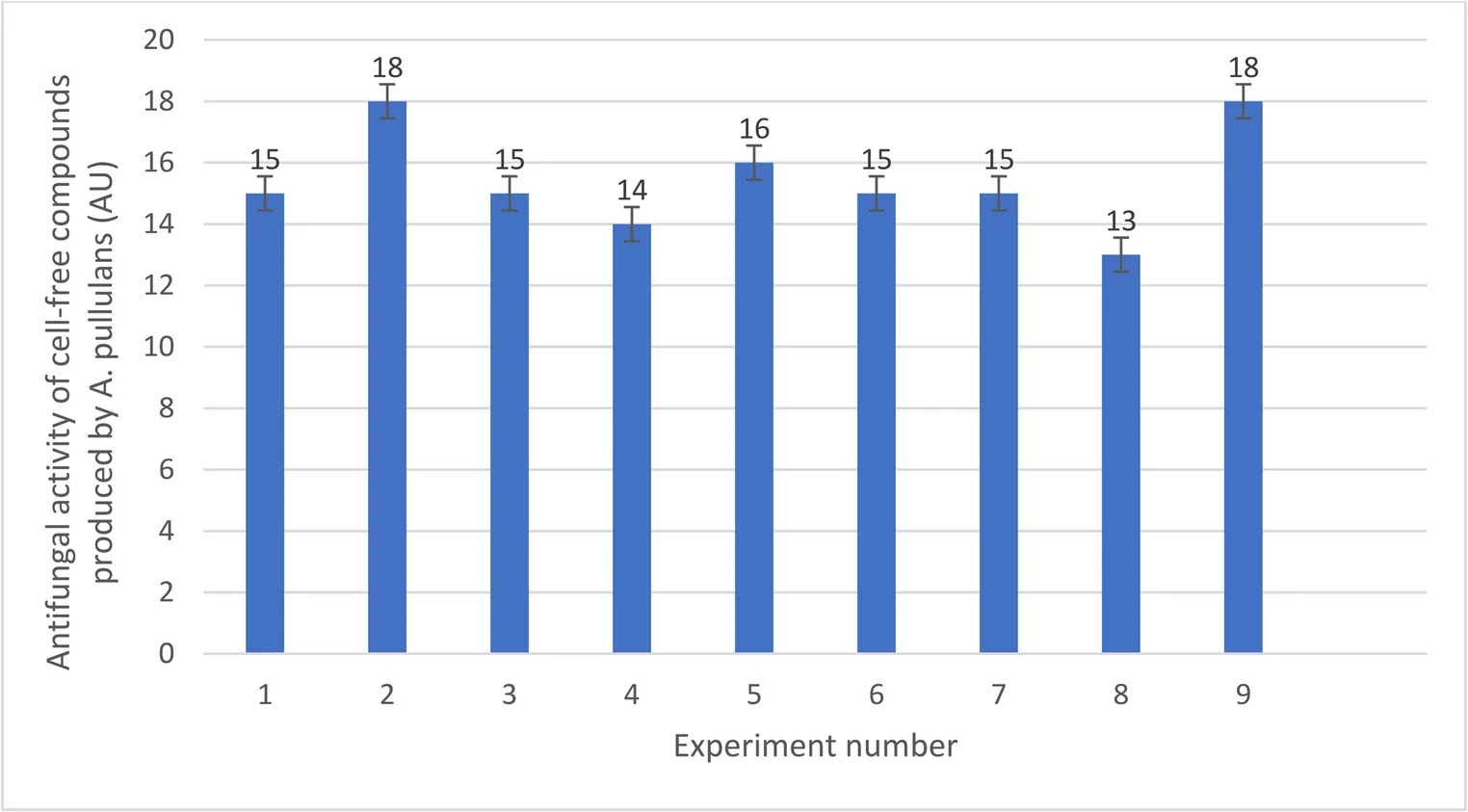
Antifungal activity (in AU) of extracellular metabolites produced by *A. pullulans* SBAN-IAUF-1 across nine experiments designed using Qualitech 4 software

In this study, the antifungal activity of extracellular metabolites produced by *A. pullulans* SBAN-IAUF-1 was evaluated in terms of AU. According to the results, experiments 2 and 9 exhibited higher antifungal activity compared with the other experiments (Fig. 5). These data were then analyzed using Qualitek 4 software. ANOVA results showed that YMB concentration and pH had the strongest effects on the production of extracellular antifungal metabolites, contributing 43% and 36.99%, respectively. In contrast, temperature and incubation time had the weakest effects, contributing only 6.9% and 13%, respectively. Degrees of freedom (DOF) for each factor equal the number of levels minus one. Sum of Squares. (S) represents the total variation attributed to the factor, while Pure Sum (S) reflects the independent contribution of each factor after removing the effects of others. F-ratio and variance were calculated to assess statistical significance. The percentage contribution shows the total amount of influence exerted by each factor (Table 4). The large F-ratio values observed in the ANOVA output were associated with the very low error variance estimated by the software. Therefore, the relative influence of the factors was primarily interpreted based on their percentage contribution values. Based on the L9 analysis, the optimal experimental conditions for maximizing antifungal activity were predicted to be 20 °C, 7 days of incubation, pH 5, and 1% YMB concentration (wv^-1^). These conditions were suggested as optimum conditions to enhance the antifungal activity of extracellular metabolites produced by *A. pullulans* SBAN-IAUF-1 (Table 5). The interaction of incubation time and pH was 60%, temperature and pH was 40%, temperature and YMB concentration was 40%, incubation time and YMB concentration was 20%, temperature and incubation time was 10%, and pH and YMB concentration was 0%. A confirmation experiment under the optimal conditions was repeated and the results on this strain showed that the antifungal activity of this strain reached 19 AU.

**Table 4:** ANOVA results showing the effects of different factors on the antifungal activity of extracellular metabolites produced by *A. pullulans* SBAN-IAUF-1

Factor	DOF	sum of Squares (S)	Variance (V)	F-Ratio (F)	Pure Sum (S)	Percent *P* (%)
Temperature	2	4.666	2.333	419992.103	4.666	6.999
Incubation time (d)	2	8.666	4.333	780015.621	8.666	13
pH	2	24.666	12.333	2219967.556	24.666	36.999
YMB concentration (% wv^− 1^)	2	28.666	14.333	2580016.822	28.666	43
Other Error	18	0.001	0.001			0.002
Total	26	66.666				100%

**Table 5:** An experiment designed by software to determine the optimal conditions for maximum antifungal activity of extracellular metabolites produced by *A. pullulans* SBAN-IAUF-1

Factor	Level Description	Level	Contribution
Temperature	20	1	0.555
Incubation time (d)	7	3	0.555
pH	5	2	1.222
YMB concentration (% wv^− 1^)	1	1	0.888

## Discussion

While indirect mechanisms such as induced resistance are important, the present study focused on direct antagonistic interactions and extracellular antifungal metabolites.

In this study, *P. commune* SBAN-IAUF-12 was reported for the first time as a fungal pathogen isolated from rotten potato tubers in Iran. Similarly, other pathogenic *Penicillium* spp., including *P. oxalicum*, *P. polonicum*, *P. sclerotigenum*, and *P. citrinum*, have been previously isolated from potatoes in countries such as South Korea, India, and Pakistan (Paul et al. 2018; Kim et al. 2008; Shah et al. 2023). These findings highlight that various *Penicillium* spp. are significant phytopathogens, posing a considerable threat to potato storage worldwide.

In general, isolating yeasts naturally present on the surfaces of leaves and fruits is advantageous, as these microorganisms are well adapted to the environmental conditions of fruits and vegetables and are generally regarded as safe. In this study, indigenous yeasts were isolated from potato leaf surfaces.

In the present study, the killer activity of yeast isolates against the fungal pathogen *P. commune* SBAN-IAUF-12 was evaluated by spotting the yeasts onto YEPD-MB medium that had been pre-inoculated with a conidial suspension of the pathogen. The medium was buffered to pH 4.3–4.7, as previous studies have shown that yeast killer activity is maximized within this pH range (Perez et al. 2016; Rosa et al. 2010; Moura et al. 2021).

In previous studies, *Saccharomyces cerevisiae* NCYC 1006 was used as a sensitive microorganism to evaluate the killer activity of yeasts against mold cells (Moura et al. 2021; Rosa et al. 2010). In the present study, the use of mold cells as the sensitive target, instead of the standard yeast strain, provided a more accurate approach for identifying killer yeasts active against the specific pathogenic mold.

In the present study, in vitro biocontrol assays showed that *A. pullulans* inhibited the growth of *P. commune* by 60%. Similarly, previous studies reported that *A. pullulans* reduced the growth of *Rhizoctonia solani* by 57% (Sanzani et al. 2021; Di Francesco et al. 2021). Overall, growth inhibition rates above 50%, as observed in both the present and previous studies, highlight the strong biocontrol potential of this yeast against pathogenic molds.

In the present study, in vivo biocontrol assays showed that *A. pullulans* reduced potato rot caused by *P. commune* by 69.41%. Similarly, previous in vivo studies have reported that different *(A) pullulans* strains decreased the incidence of pathogenic grape molds (Schena et al. 2003) and reduced cherry and tomato rots caused by *(B) cinerea* and *Phytophthora infestans* by 47% and 32%, respectively (Di Francesco et al. 2017). Additional in vivo experiments showed that *A. pullulans* reduced *Geotrichum citri-aurantii* rot in oranges by 30% (Ferraz et al. 2016). Another study demonstrated that an *A. pullulans* strain decreased rot caused by *R. solani* in bean and soybean plants by 80% and 50%, respectively (Di Francesco et al. 2021). Overall, the antifungal activity of *A. pullulans* SBAN-IAUF-1 observed in the present study is consistent with the biocontrol performance reported for other *A. pullulans* strains in previous research.

In this study, the antifungal activity of extracellular metabolites produced by *A. pullulans* SBAN-IAUF-1 was optimized for the first time using the Taguchi method, focusing on key experimental parameters including temperature, incubation time, pH, and YMB concentration. Furthermore, previous studies optimized the culture medium for biomass production of *Rhodosporidium paludigenum*, a biocontrol yeast, using the Plackett–Burman design (Wang et al. 2011). In those cases, the optimization aimed to enhance yeast biomass, whereas in the present study, the optimization was conducted to improve the antifungal activity of the yeast using the Taguchi statistical approach. After conducting the nine experiments designed by the software, the optimal conditions for producing extracellular antifungal metabolites by *A. pullulans* SBAN-IAUF-1, based on the L9 analysis, were identified as 20 °C, a 7-day incubation period, pH 5, and 1% YMB medium (wv^-1^). Based on ANOVA results, YMB medium concentration and pH had the highest effects at 43% and 36.99%, respectively, while temperature and incubation time had the lowest effects at 6.9% and 13%, respectively, on the production of extracellular antifungal metabolites by this yeast. The large F-ratio values observed in the ANOVA output were associated with the very low error variance estimated by the software (0.002% contribution). In previous work, optimization of pH enhanced the biocontrol activity of *Wickerhamomyces anomalus* against the wine spoilage yeast *Zygosaccharomyces rouxii* (Kuchen et al. 2024). Consistent with these findings, the software analysis in the present study also indicated that pH plays a significant role in regulating extracellular antifungal metabolites production. Therefore, the results of this study are in agreement with earlier reports. In general, temperature is a key factor influencing the production of extracellular antifungal metabolites by yeasts. In a previous study (Corbaci and Ucar 2017), the impact of temperature on extracellular antifungal metabolites production was evaluated at 10, 15, 20, 25, and 30 °C, and the results showed that the yeast exhibited its strongest antifungal activity at 20, 25, and 30 °C, respectively. Accordingly, in the present study, extracellular antifungal metabolites production was assessed at three temperature levels: 20, 25, and 30 °C. The L9 analysis revealed that 20 °C was the most favorable temperature for enhancing extracellular antifungal metabolites production in this yeast. These findings are therefore consistent with previous reports. Incubation time is also an important factor influencing the production of extracellular antifungal metabolites by yeasts. In the present study, incubation periods of 3, 5, and 7 days were selected as experimental levels. In a study (Bajaj et al. 2013), the killer activity of a yeast strain was assessed by co-culturing a killer yeast with a sensitive strain for 5 days. The results showed a gradual decline in the number of sensitive cells from day 2 to day 5, reaching the lowest level on the fifth day. In the present study, the software analysis demonstrated that the highest level of extracellular antifungal metabolites production occurred on day 7. The results of this study, along with previous reports, suggest that longer yeast incubation times result in increased production of extracellular antifungal metabolites. pH is also regarded as a critical factor in the production of extracellular antifungal metabolites by yeasts. The Taguchi analysis in the present study indicated that pH 5 was the optimal condition for maximizing extracellular antifungal metabolites production in this yeast. In a previous study (Agirman and Erten 2020), extracellular antifungal metabolites produced by an *A. pullulans* strain were evaluated at pH levels 4 and 6. Accordingly, pH values of 4, 5, and 6 were selected as appropriate levels in the present investigation. In this study, YMB medium was used based on findings by Corbaci and Ucar (2017), who compared extracellular antifungal metabolites production in various media, including PDB (Potato dextrose broth), YCB (Yeast carbon base broth), YMB (Yeast malt broth), YNB (Yeast nitrogen base broth), and YEPD (Yeast extract, peptone, glucose broth). Their results showed that YMB supported higher extracellular antifungal metabolites production than the other tested media. In general, the quantity of food sources is an important factor in the production of extracellular antifungal metabolites by yeast. Therefore, in this study, different concentrations of the yeast culture medium (YMB) were evaluated as an important parameter, using levels of 1, 1.5, and 2% (wv^-1^). The Taguchi analysis showed that the highest level of extracellular antifungal metabolites production occurred at the 1% concentration, which was the lowest nutrient level tested. This may be explained by the yeast entering the stationary phase under nutrient-limited conditions, during which secondary metabolites, including extracellular antifungal metabolites, are typically produced. The strong antifungal activity in the 1% YMB treatment suggests that *A. pullulans* SBAN-IAUF-1 produces more secondary metabolites when nutrients are limited. Yeasts often increase the secretion of stress-related antimicrobial metabolites in poor media, which likely explains the higher activity observed here.

Previous studies have investigated the antifungal activity of *A. pullulans* against various *Penicillium* species, such as *P. expansum* and *P. digitatum*, in apples and pears (Mounir et al. 2007; Agirman and Erten 2020; Ferreira-Pinto et al. 2006). However, this is the first report demonstrating the biocontrol activity of *A. pullulans* against *P. commune*.

The new strain *A. pullulans* SBAN-IAUF-1, isolated from the unique potato agroecosystem of Iran, represents a previously unstudied yeast resource. Its strong activity against *P. commune* highlights its potential as a practical, low-cost, and environmentally friendly biocontrol option. This locally adapted isolate may contribute to developing more effective and region-specific postharvest management strategies.

Additionally, Schena et al. (2003), Mari et al. (2012), Di Francesco et al. (2017), Klein and Kupper (2018), Galli et al. (2021), and Di Francesco et al. (2021) examined the biocontrol efficacy of *A. pullulans* against fungal rots in cherries, apples, tomatoes, citrus fruits, grapes, and cereals, respectively. To date, however, no studies have reported antifungal activity assays of *A. pullulans* against fungal diseases in potatoes.

Despite the promising results, this study has several limitations. First, the specific antifungal compounds responsible for the observed activity were not identified, and further chemical characterization is required. Second, although evaluating extracellular antifungal metabolites on potato tubers would provide valuable additional insights, this experiment was beyond the scope of the present study. Third, the experiments were conducted under controlled laboratory conditions, and validation under greenhouse and field conditions is necessary to confirm the practical applicability of the findings. Finally, the study focused on direct antagonistic activity, while other mechanisms, such as induced resistance, were not investigated. Future studies should address these aspects to provide a more comprehensive understanding of the biocontrol potential of *Aureobasidium pullulans*.

## Conclusion

In this study, *Aureobasidium pullulans* SBAN-IAUF-1 effectively inhibited the growth of *P. commune* SBAN-IAUF-12, an important postharvest potato pathogen, mainly through extracellular antifungal metabolites. Optimization using the Taguchi method further enhanced the antifungal activity of these metabolites. The findings indicate that *A. pullulans* SBAN-IAUF-1 is a promising, sustainable, and environmentally friendly alternative to chemical fungicides for managing postharvest diseases in potatoes and other crops. Overall, this yeast shows strong potential for commercialization as a biocontrol agent against postharvest fungal pathogens.

## Data Availability

Data availability statementThe nucleotide sequence data generated in this study have been deposited in the GenBank database under accession numbers OR626047 and PP704689.1. All other data supporting the findings of this study are available within the article.
